# Cannabinoid CB2 Receptors in a Mouse Model of Aβ Amyloidosis: Immunohistochemical Analysis and Suitability as a PET Biomarker of Neuroinflammation

**DOI:** 10.1371/journal.pone.0129618

**Published:** 2015-06-18

**Authors:** Alena V. Savonenko, Tatiana Melnikova, Yuchuan Wang, Hayden Ravert, Yongjun Gao, Jeremy Koppel, Deidre Lee, Olga Pletnikova, Eugenia Cho, Nuzhat Sayyida, Andrew Hiatt, Juan Troncoso, Peter Davies, Robert F. Dannals, Martin G. Pomper, Andrew G. Horti

**Affiliations:** 1 Department of Pathology, The Johns Hopkins University School of Medicine, Baltimore, MD, United States of America; 2 Departments of Neurology, The Johns Hopkins University School of Medicine, Baltimore, MD, United States of America; 3 Russell H. Morgan Department of Radiology and Radiological Science, The Johns Hopkins University School of Medicine, Baltimore, MD, United States of America; 4 Litwin-Zucker Research Center, Feinstein Institute for Medical Research, North-Shore Long Island Jewish Health System, Manhasset, NY, United States of America; 5 MAPP Biopharmaceutical Inc, San-Diego, CA, United States of America; Massachusetts General Hospital and Harvard Medical School, UNITED STATES

## Abstract

In Alzheimer’s disease (AD), one of the early responses to Aβ amyloidosis is recruitment of microglia to areas of new plaque. Microglial receptors such as cannabinoid receptor 2 (CB2) might be a suitable target for development of PET radiotracers that could serve as imaging biomarkers of Aβ-induced neuroinflammation. Mouse models of amyloidosis (J20APPswe/ind and APPswe/PS1ΔE9) were used to investigate the cellular distribution of CB2 receptors. Specificity of CB2 antibody (H60) was confirmed using J20APPswe/ind mice lacking CB2 receptors. APPswe/PS1ΔE9 mice were used in small animal PET with a CB2-targeting radiotracer, [^11^C]A836339. These studies revealed increased binding of [^11^C]A836339 in amyloid-bearing mice. Specificity of the PET signal was confirmed in a blockade study with a specific CB2 antagonist, AM630. Confocal microscopy revealed that CB2-receptor immunoreactivity was associated with astroglial (GFAP) and, predominantly, microglial (CD68) markers. CB2 receptors were observed, in particular, in microglial processes forming engulfment synapses with Aβ plaques. In contrast to glial cells, neuron (NeuN)-derived CB2 signal was equal between amyloid-bearing and control mice. The pattern of neuronal CB2 staining in amyloid-bearing mice was similar to that in human cases of AD. The data collected in this study indicate that Aβ amyloidosis without concomitant tau pathology is sufficient to activate CB2 receptors that are suitable as an imaging biomarker of neuroinflammation. The main source of enhanced CB2 PET binding in amyloid-bearing mice is increased CB2 immunoreactivity in activated microglia. The presence of CB2 immunoreactivity in neurons does not likely contribute to the enhanced CB2 PET signal in amyloid-bearing mice due to a lack of significant neuronal loss in this model. However, significant loss of neurons as seen at late stages of AD might decrease the CB2 PET signal due to loss of neuronally-derived CB2. Thus this study in mouse models of AD indicates that a CB2-specific radiotracer can be used as a biomarker of neuroinflammation in the early preclinical stages of AD, when no significant neuronal loss has yet developed.

## Introduction

Alzheimer's disease (AD), which affects more than 5.4 million individuals in the United States [1], is characterized by progressive deficits in memory, cognition and behavior that ultimately lead to dementia [2, 3]. Neuropathological hallmarks of AD include the extracellular deposition of β-pleated assemblies of Aβ peptide, forming senile plaques, and intracellular aggregates of hyperphosphorylated tau protein, forming neurofibrillary tangles [4]. The pathophysiological processes that underlie AD begin decades before clinical symptoms [5] with biomarkers of Aβ amyloidosis changing 15–25 years before the onset of clinical symptoms [6]. Such long prodromal stages of the disease provide an opportunity for early diagnosis and treatment if biomarkers reflective of early pathophysiological processes are available.

An early response to Aβ deposition is recruitment of microglia to areas of a new plaque, which occurs within 24–48 hours of plaque deposition [7]. The localized microglial response to fibrillar Aβ is characterized by upregulation of toll-like, prostanoid, and complement receptors [8, 9] as well as de novo synthesis of other cell-surface receptors such as cannabinoid receptor 2, CB2 [10, 11]. One or more of those microglial receptors might be a suitable target for development of a positron emission tomography (PET) radiotracer that serves as an imaging biomarker of Aβ -induced neuroinflammation. A number of CB2 radioligands have been synthesized by several research groups (see for review [12]). Most published CB2 imaging studies describe substantial binding of radiotracer to the spleen and discuss the ability of the radioligands to cross the blood-brain barrier as well as aspects of non-specific binding in control rather than models relevant to AD. Whether these PET radiotracers can serve to study neuroinflammation, and particularly neuroinflammation in the setting of AD [13], is still unclear.

Researchers from KU Leuven University used animals that were stereotactically injected with viral vectors that drove expression of human CB2 receptors in the striatum [14–16]. In this condition, PET imaging revealed specific and reversible binding of CB2 radiotracers to overexpressed human CB2 receptors [14–16]. The best radioligand of this series, [^11^C]NE40, was studied in healthy human subjects [17], but [^11^C]NE40 did not demonstrate an increase of CB2 binding in AD patients versus healthy controls [18].

The most recent CB2 radioligand, developed by the Sanofi and MNI groups, demonstrated substantial specific binding (40–50%) in the control baboon brain and an increase in cerebral binding in the baboon that was treated with the endotoxin, lipopolysaccharide (LPS) [19].

Abbott Laboratories has recently synthetized a selective CB2 agonist, A836339, with very high CB2 binding affinity and excellent CB2/CB1 selectivity [20]. We radiolabelled A836339 with ^11^C and studied [^11^C]A836339 in vivo [21]. By using two animal models of neuroinflammation, a lypopolysaccharide (LPS)-induced mouse model [22] and a transgenic amyloid mouse model of AD (APPswe/PS1ΔE9 mice) [23, 24], we observed substantial specific binding of [^11^C]A836339 in the dissected cortex and hippocampus [21]. The specific binding of [^11^C]A836339 in the brains of LPS-treated mice was in accordance with previously described upregulation of CB2 receptors in this model of neuroinflammation [25]. This observation might be relevant to and consistent with findings from brain tissues from patients with AD, which showed increased expression of CB2 receptors in areas of Aβ amyloid plaque deposition [26]. However, detailed histological studies of CB2 receptor distribution in mouse models of AD are lacking.

Here we use APPswe/PS1ΔE9 mice to characterize the distribution of CB2 receptors in different cells of the brain and assess suitability of this receptor as a PET biomarker of neuroinflammation. An additional APP transgenic model, J20 APPswe/ind [27] [28], is used to characterize specificity of CB2 antibody by crossing them to mice that lack CB2 receptor. The APPswe/PS1ΔE9 model used in PET studies reproduces important features of AD including elevated levels of Aβ (particularly more amyloidogenic Aβ1–42 peptide); aging-related accumulation of amyloid plaques; reductions in neurotransmitter markers; age-related and Aβ-dependent cognitive impairments [24, 29–31]. However, as most other APP mouse models of AD, this model does not reproduce significant tau pathology or loss of neurons. Combination of these features supports a view that even very old APP mice only appear to be good models of early stages AD [32]. Nevertheless, both the APPswe/PS1ΔE9 and J20 APPswe/ind mice recapitulate microglial activation and reactive gliosis induced by fibrillar and/or oligomeric forms of Aβ [33] [7, 34–37], supporting the validity of these models for study of neuroinflammation.

## Materials and Methods

### Animals

This study was conducted according to NIH guidelines for animal care. All procedures for APPswe/PS1Δ9 mice were approved by the Johns Hopkins University School of Medicine Animal Care and Use Committee. All experiments in J20/CNR2 mice were performed according to procedures approved by the Feinstein Institute for Medical Research Institutional Animal Care and Use Committee.

The PET experiments and immunohistological studies were performed at JHU using APPswe/PS1ΔE9 bigenic transgenic mice, Line 85 [23, 24] (strain name: B6.C3-Tg(APPswe,PSEN1dE9)85Dbo/Mmjax; Jackson Laboratory, Bar Harbor, USA) backcrossed to congenic C57BL/6J background for more than 15 generations. APPswe/PS1ΔE9 mice express chimeric mouse/human amyloid precursor protein (APP) (Mo/HuAPP695) with the double Swedish mutation (K595N/M596L) and mutant human Presenilin 1 lacking exon 9 (PS1ΔE9) under control of mouse Prion promoter [23, 24]. Male and female double transgenic mice were used and compared to their age- and sex-matched non-transgenic littermates (NTG). All mice had free access to food and water and were housed in automatically controlled light conditions (light 7 am-9 pm). Animals were kept under these conditions until they were taken to the small animal PET testing room, at least 2 hrs before the experiments, or before sacrifice.

An additional APP transgenic model, J20 APP [27] [28], was used in histological studies. J20 APP mice (Strain name B6.Cg-Tg(PDGFB-APPSwInd)20Lms/2Mmjax) express human APP with the double Swedish and Indiana mutations under control of the human platelet–derived growth factor β polypeptide promoter [27] [28]. These mice were crossed with CB2 knockout mice lacking C-terminus amino acid positions 217 to 347 (strain name B6.129P2-Cnr2tm1Dgen) [38] to receive J20/CNR2-/- and J20/CNR2+/+ mice and were used for testing the specificity of an anti-C-terminal CB2 antibody (H60, SantaCruz). Paraffin slides from the brains of J20;CNR2-/- and J20;CNR2+/+ mice were kindly provided by Drs. Koppel and Davies (The Feinstein Institute for Medical Research).

### Human tissue

Human AD and age-matched cognitively-normal brain samples were obtained from the Johns Hopkins Medical Institution Brain Resource Center (http://www.ninds.nih.gov/research/parkinsonsweb/brain_banks/brain_biospecimen_repositories.htm). The brains were examined at the Division of Neuropathology, Johns Hopkins University and clinical diagnoses were confirmed by neuropathological examination. Tissue sections from right hippocampus were used in this study.

### CB2, NeuN, CD68, and GFAP immunofluorescent staining

The immunofluorescence staining was performed to study the presence of CB2 receptors and their colocalization with neuronal (NeuN antibody), microglial (CD68) or astroglial (GFAP) markers. The procedures were slightly modified from previous methods [39]. Groups of 3–4 APPswe/PS1ΔE9 mice and their control littermates were perfused intracardially with cold phosphate-buffered saline (pH 7.6; PBS) under deep anesthesia. The brains were removed, cut sagittally in a half. The left hemispheres were fixed by an immersion in 4% paraformaldehyde in PBS at 4°C overnight, the hemispheres were washed with several changes of PBS, went through 15% and 30% sucrose solution in PBS for cryoprotection, snap frozen in isopentane on dry ice, and stored at -80oC. Sagittal sections (30 μm) were made on a freezing sliding microtome and stored in anti-freeze buffer at -200 C until use. Immunofluorescent staining with primary antibodies was performed on free floating sections. After washing with PBS, sections were blocked for 1 hr at room temperature (RT) in the blocker solution containing 3% normal goat serum, 3% BSA (Sigma, USA), and 0.3% Triton X-100 in PBS, followed by incubation with primary antibodies prepared in blocker. The incubation was started at RT for 2 hrs and then continued at 4°C overnight. After thorough washing in PBS, sections were mounted on the slides prior to application of secondary antibodies. Each slide included sections from both non-transgenic and AD animals and incubated in a mixture of secondary antibodies for 1 hr in RT. Before mounting slides with anti-faded mounting media (Invitrogen), sections were counterstained with 4',6' diamino-2-phenylindole·2HCl (DAPI). To control for non-specific labeling, the adjacent brain sections from AD mice were incubated only with secondary antibodies.

The frozen sections from human brains were processed the same way as mouse frozen sections described above. Paraffin slides from J20/CNR2-/- and J20/CNR2+/+ mice were from the brains fixed with 4% paraformaldehyde in 0.1 molar PBS. After deparaffination of sections with xylene and rehydration in ethanol and water, antigen retrieval was done by heating sections in 10 mM sodium citrate buffer. We used 2% nonfat milk in PBS for blocking a non-specific binding and for antibody preparations.

### Antibodies

The following primary antibodies were used in different combinations: anti-CB2 receptor antibodies raised against C-terminal amino acids 301–360 (Santa Cruz H60; 1:300) or N-terminal amino acids 20–33 (Cayman Chemical # 101550; 1:300), CD68 (AbD Serotec; 1:400), Neu-N (Millipore, 1:400), GFAP (rbGFAP Dako or mGFAP-71.1 Sigma; 1:400), and corresponding goat anti-mouse, anti-rabbit and anti-rat Dylight 549, 633, and 488-conjugated secondary antibodies (Jackson ImmunoResearch Lab, Inc, 1:400).

The main CB2 antibody used in our study (the C-terminal rabbit CB2R antibody from Santa Cruz) has been previously validated by the lab of Dr. George Uhl (NIH) [40]. The pre-adsorption and co-incubation of the CB2-R antibody with the immunizing peptide were performed resulting in blocking CB2 immunostaining in the rat cerebellum. Western blot analyses from mice brains revealed a major CB2-R band of approximately 53 kDa, with other visible bands around 37 kDa and 75 kDa, similar to those reported by Van Sickle et al. [41]. Cayman antibody (the N-terminal rabbit CB2R antibody) has been previously verified for sensitivity and specificity [42–44]. Similar to the Santa Cruz antibody, Cayman antibody revealed three CB2-R bands (37, 44 and 59 kDa) when tested by Western blot of rat tissues [43]. Specificity of Cayman antibody was confirmed in HEK cells transiently transfected with human CB2 [44] and in CB2 knockout mice with deletion of the first three N-terminal domains [42].

### Analyses of immunofluorescent images

Images from sections were first obtained using an epifluorescence Zeiss Axiophot microscope with SPOT Imaging Advanced software. For each genotype and animal, two-three sections were evaluated with lateral localization, 0.7–1.5 mm from a midline. For a more detailed analysis of multiple immuno costainings, the slides were examined using a confocal Zeiss Axiovert 200 microscope with a Zeiss LSM510-Meta confocal module. Four lasers were used with excitation wavelengths of 405 (diode laser; blue channel), 488 (argon laser; green channel), 542 (green HeNe; red channel), and 633 nm (red HeNe; far-red channel). The images were taken as Z-stacks in Zen software, saved in LSM format, and analyzed with an open-source image processing package Fiji ([Fiji Is Just] ImageJ) [45]. To correct illumination artifacts, the images were processed using a “Subtract background” command. To characterize the intensity of staining along a region of interest, a “Plot Profile” command was used. To correct for autofluorescence of cytoplasmic aggregates in neurons, intensity on DAPI channel was thresholded to highlight the cytoplasmic aggregates that were excluded from a region of interest (ROI) by using ROI manager in Fiji software. To analyze the intensity of CB2 receptor staining in marker-positive (ROI) and-negative areas, a marker’s channel was processed by a threshold function to create a selection for marker-positive and-negative areas; then, average signal intensities for both areas were measured on a CB2 channel. 3D image reconstruction was performed using Imaris x 64 7.4.2 software (Bitplane USA, South Windsor, CT).

For construction of figures with multiple panels, files were exported in TIF format to Photoshop 13.0.1 x 64 software (Adobe Systems Inc.). To target images for better printing/viewing, tonality was adjusted by increasing brightness of midtones by Levels, Curves and/or Exposure commands. Adjustments for images from mice of different genotypes and conditions were kept the same. Adjusted images were not used for analyses of staining intensities.

### Radiosynthesis of [^11^C]A836339

[^11^C]A836339 (2,2,3,3-tetramethylcyclopropanecarboxylic acid [3-(2-[^11^CH_3_]methyloxyethyl)-4,5-dimethyl-3*H*-thiazol-(2*Z*)-ylidene]amide) was synthesized as described previously [21]. In brief, no-carrier-added [^11^C]methyl triflate was synthesized by reaction of silver triflate with [^11^C]methyl iodide that was prepared from [^11^C]CO_2_ using a Tracerlab FX MeI module (General Electric) and a PETtrace biomedical cyclotron (General Electric). [^11^C]Methyl triflate was heated in a solution of dimethylformamide with nor-methyl-A836339 and appropriate base. [^11^C]A836339 was purified by semi-preparative high-performance liquid chromatography (HPLC) and formulated as a sterile, apyrogenic solution in normal saline with 8% ethanol with a specific radioactivity of 17,200 ± 5,400 mCi/μmol (n = 11) and radiochemical purity >95%.

### Small animal PET experiments

#### Baseline

A dedicated small animal PET scanner (eXplore VISTA; GE Healthcare) and small animal CT scanner (X-SPECT/CT; Gamma Medica) were used. In all experiments, the PET session was conducted for two mice simultaneously with the same batch of [^11^C]A836339. One mouse from a pair represented a control group (NTG or no-treatment group) whereas the other mouse represented an experimental group (transgenic or treatment group). Mice were induced and anesthetized with isoflurane. Dynamic PET scans were acquired for 30 min (20 sec x 3, 30 sec x 2, 1 min x 2, 2 min x 3, 5 min x 4) immediately after an intravenous bolus injection of [^11^C]A836339 (0.11–0.14 mCi, specific radioactivity 8,600 ± 2,100 mCi/μmol). A CT scan was acquired shortly after the PET scan as a reference for localization of brain regional radiotracer uptake through PET-CT image fusion performed off-line. In all PET experiments, a 250–700 keV energy window was used, and the data were reconstructed using an iterative 2D ordered-subject expectation-maximization method, using a trans-axial pixel size of 0.4 mm and axial slice thickness of 0.8 mm [46]. No attenuation and scatter corrections were applied, as they have relatively small impact on mouse brain imaging.

#### CB2 binding specificity

In vivo CB2 binding specificity (blocking) PET scans were carried out by subcutaneous (s.c.) administration of 2 mg/kg of AM630 (Tocris, Bristol, UK), a CB2 selective inverse agonist, followed by i.v. injection of [^11^C]A836339 15 min thereafter, similar to the baseline experiments above. The blocker was dissolved in a water-dimethylsulfoxide (DMSO) mixture (1:1) and injected in a volume of 0.1 mL. Control animals were injected with 0.1 mL of the vehicle.

#### In vitro binding assay of A836339 and NE40

The assay was done in duplicate commercially by CEREP (France) using stably transfected CHO cells with human CB2 receptor and a radioligand, [^3^H]WIN55212-2, as previously described [47].

### PET image analysis

Image analysis was performed using the software package PMOD (v3.3, PMOD Technologies Ltd). For each study, first, the reconstructed PET and CT brain images were co-registered; next, the existing Mouse Brain template volumes of interest (VOIs) [48, 49] were “morphed” to match the brain image of the fused PET-CT; finally, the pre-defined VOIs were then applied to the dynamic PET data to generate time-activity curves (TACs) as well as regional standardized uptake values [SUV = (tissue radioactivity concentration/injected dose) x body weight] in selected time windows. Those TACs and SUVs were subsequently compared between different animals (e.g., control vs. AD mice).

### Statistical analyses

The immunofluorescence data of staining intensities were analyzed using analyses of variance (ANOVA) with the statistical package STATISTICAx64 10 (StatSoft, Inc., Tulsa, OK) and a minimal level of significance for effects of interactions p<0.05. One-way ANOVA was used for comparisons of staining intensities in different regions of interest (ROI) with background. One-way ANOVA was also used for analysis of plot profile intensities at different distances from a core of Aβ plaque. Two-way ANOVA was utilized to analyze CB2 staining intensities in different groups of mice (non-transgenics vs AD mice) and ROIs (NeuN-, CD68-, and GFAP- positive areas). LSD post-hoc tests were applied to a significant Group x ROI interaction. One-way ANOVA was used for comparisons of PET experiments.

## Results

### CB2 receptors are expressed in neurons of the AD mouse model and AD human cases

Our expectation that an AD mouse model would have CB2 receptors expressed by neurons was based on earlier findings of such expression in non-transgenic mice/rats, as well as in models of other neurodegenerative diseases and neuroinflammation [40, 50, 51] [52] [53].

**Fig 1:**
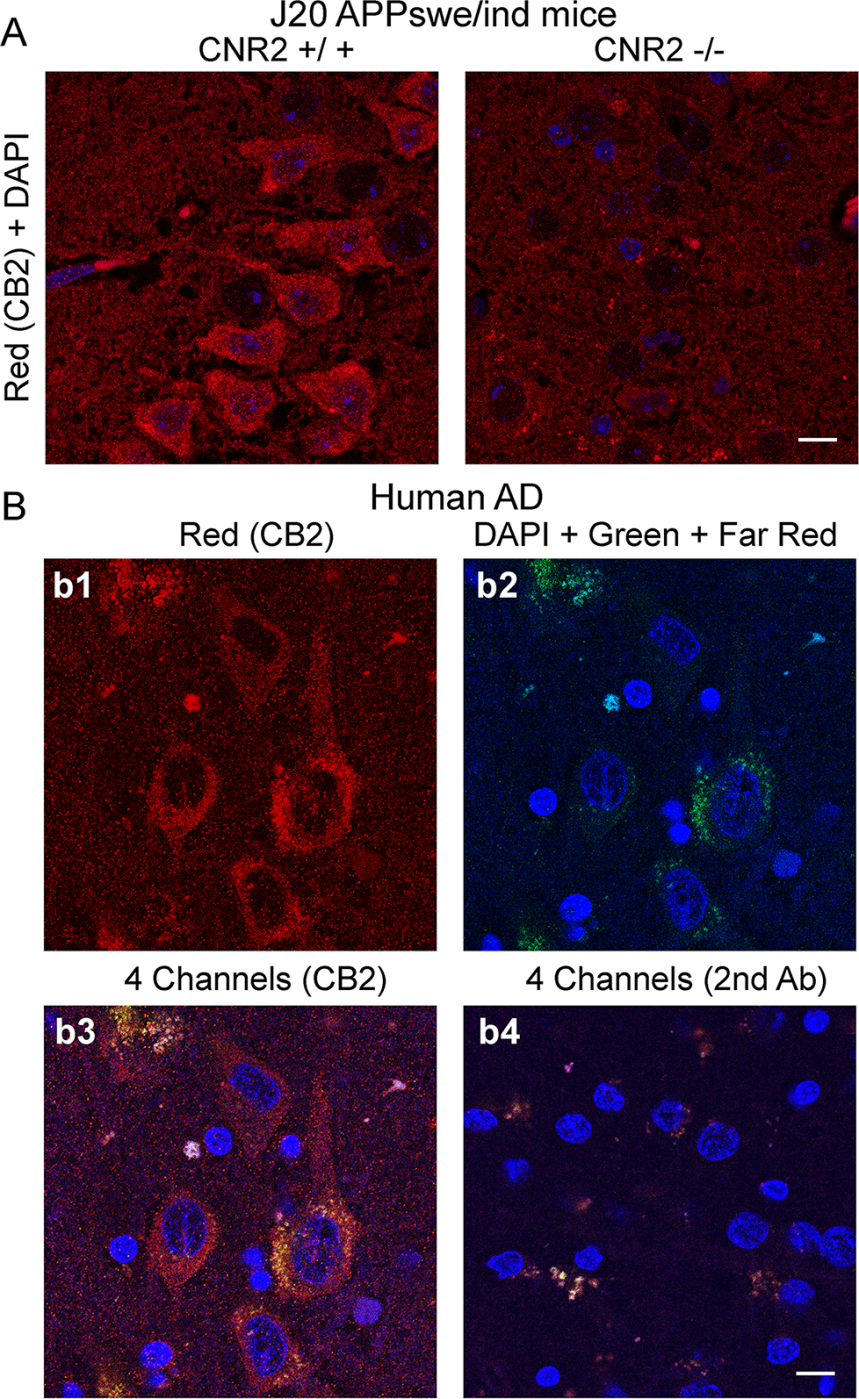
Expression of CB2 receptors in neurons of the hippocampus in a mouse model of amyloidosis (12-mo old J20 APP mice) (A) and in a patient with Alzheimer’s disease (B). (A) Representative confocal images of double immunostaining using DAPI (blue) and CB2 R antibody (H60, Santa Cruz) (red). CA3 area of the hippocampus is shown. To check for specificity of H60 antibody J20 mice were used that either have both (left) or no copies (right) of a CNR2 gene that encodes for CB2 receptor. (B) Confocal images of CB2 staining (b1: red, H60) in neurons of the hippocampus from postmortem brain of a patient with AD. Blue channel was used for detection of DAPI staining (b2: blue). Note the presence of some fluorescent aggregates localized in perinuclear areas of big neurons (shown as pseudo green). These aggregates were visible through different channels (blue, green and far red) suggesting that autofluorescence could be a possible source of these signals. Green and far red channels were used to detect these non-specific signals (b2). b3 shows the composite of b1 and b2. b4 shows the composite of the four channels for an image stained only with secondary antibody. Note the presence of fluorescent granules positive on all channels (white color). Scale in A and B is 10 μm.

Using brain slices of 12-mo old J20 APP mice we performed CB2 immunostaining with H60 antibody and DAPI counterstaining. Noticeable accumulation of CB2 signal was observed in neuronal cytoplasm and some dendrites as shown for pyramidal neurons of CA3 area of the hippocampus (Fig 1A). In J20APP mice lacking CB2 receptor (J20;CNR-/- mice) neuronal staining was on the level of background (Fig 1A, right panel). Mice lacking CB2 receptor, however, revealed the presence of some fluorescent aggregates localized in perinuclear areas of big neurons. These aggregates were visible through different channels (green and far red, not shown) suggesting that the autofluorescence could be a possible source of these signals. Similar pattern of staining was observed in hippocampal neurons of 12-mo old APPswe/PS1ΔE9 mice (S1 Fig).

In slices of the hippocampus in human cases with AD, H60 CB2 antibody revealed cytoplasmic and some dendritic staining in neurons (Fig 1B) similar to that in the AD mouse model (Fig 1A). Small aggregates with non-specific staining were observed over wide range of spectrum and were present on different channels after staining with only secondary antibody (Fig 1B) confirming autofluorescent nature of this signal in aged brain tissue. Our attempts to decrease the autofluorescence from cytoplasmic aggregates by pretreating the slices with Sudan Black unfortunately resulted in overall weak immunostaining (not shown). For the purposes of quantification of neuronal CB2 staining, the autofluorescence signal was then corrected by masking the DAPI-positive cytoplasmic granules.

**Fig 2:**
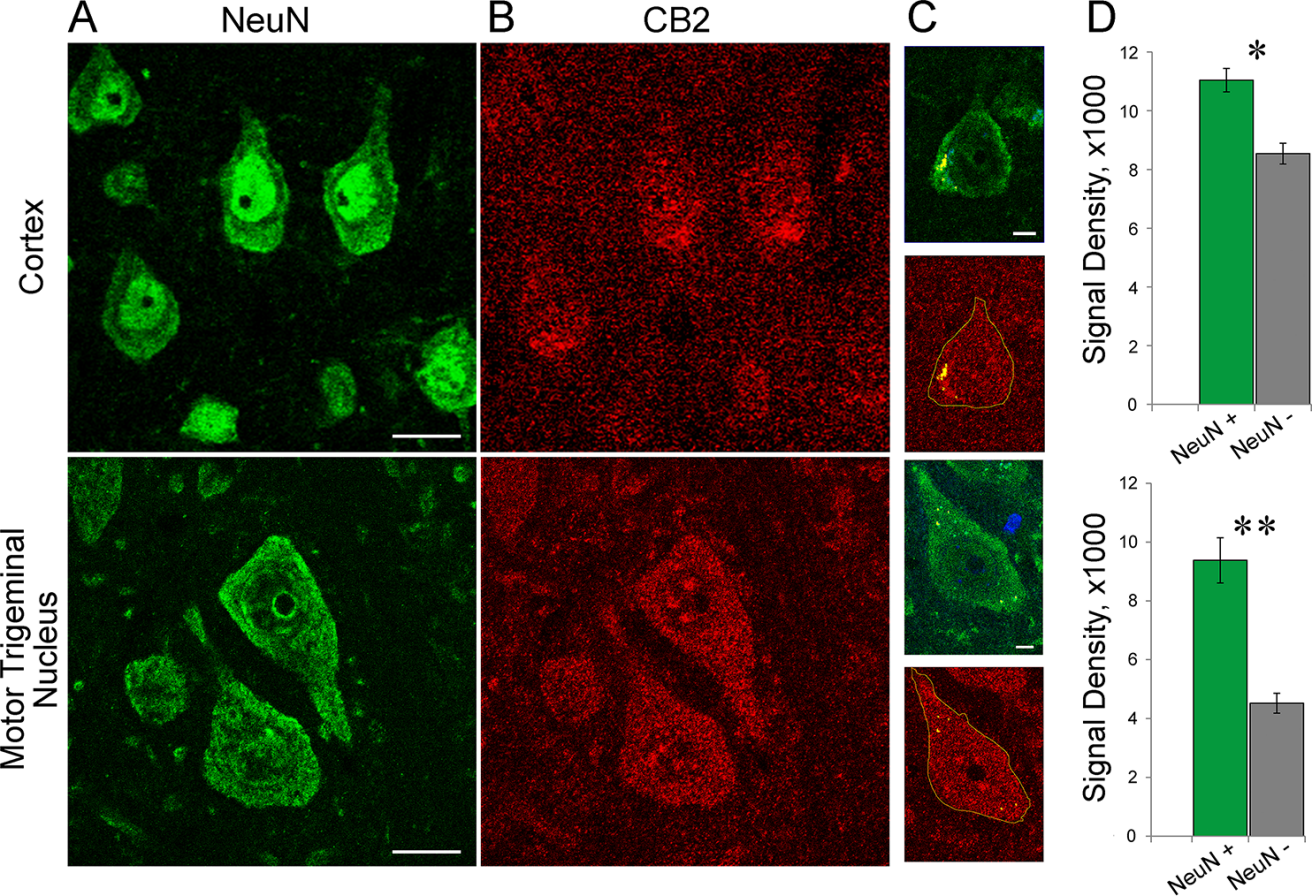
CB2 immunoreactivity in neurons of the cortex and motor trigeminal nucleus of 12 mo-old APPswe/PS1ΔE9 transgenic mice. Representative confocal images of cortical neurons (upper panels) and neurons of the motor trigeminal nucleus (lower panels) with double immunostaining using NeuN (green; A) and CB2 R (red, H60: B) primary antibodies. Scale is 15 μm. C. Example of neuronal bodies visualized by NeuN (green). The neuron outlines were transferred to CB2 channel (red). Note cytoplasmic granules (pseudo-yellow) with autofluorescence visible on DAPI channel (not shown). The areas occupied by such granules were not considered in the analyses of CB2 immunoreactivity. Scale is 5 μm. D. Quantification of CB2 densities (integrated intensities/area) in NeuN-positive and –negative areas. Signal intensities were averaged across 30 non-overlapping fields (n = 2 mice). * and ** indicate significant differences in CB2 signal between NeuN-positive areas and background, p<0.01 and 0.001, respectively (ANOVA).

Similar pattern of staining was observed in cortical neurons of 12-mo old APPswe/PS1ΔE9 mice (Fig 2). After correction for autofluorescence, analyses of CB2 immunoreactivity confirmed a significantly increased CB2 signal in NeuN positive areas as compared to NeuN negative areas (Fig 2D). In addition to the anti-CB2 antibody described so far (Santa Cruz H60 raised against C-terminus of CB2), we used a second anti-CB2 antibody raised against N-terminus of CB2 (Cayman # 10155). The immunospecificity of latter was confirmed in the mice lacking the N-terminus of CB2 receptor [41]. The immunostaining of APPswe/PS1ΔE9 brains with Cayman antibody revealed CB2 immunoreactivity in cytoplasm of pyramidal neurons similar to H60 antibody and more pronounced staining of dendrites (S2 Fig). Both antibodies resulted in relatively high background staining in cortical and hippocampal areas. To confirm that insufficient optimization of staining protocols was not a reason for low discrimination, we used the same slices and quantified neuronal CB2 signal in the motor trigeminal nucleus. This nucleus served as an example of the first areas of the CNS (brainstem nuclei) where presence of neuronal CB2 receptors was detected [41]. Differences in CB2 immunoreactivity between NeuN positive—and NeuN negative-areas were much more dramatic in the brainstem nucleus than in the cortex mainly due to lower background in the former (Fig 2). This finding indicated that the relatively high background of CB2 staining in the cortex as well as in the hippocampus is likely a characteristic of the structure (possibly to due richer neuropil) rather than due to insufficient optimization of the staining protocols.

### CB2 receptors are expressed by microglia of the AD mouse model

It is well documented that CB2 receptors are abundantly expressed in peripheral immune tissues [54–57]. There are also a number of observations reporting increased CB2 expression in plaque-associated microglia in AD brains and brains of patients with Down’s syndome-associated Aβ amyloidosis [26, 58].

APPswe/PS1ΔE9 transgenic mice showed CB2 immunolabeling consistent with these findings (Fig 2). As shown in representative epifluorescent images of CA3 areas of the hippocampus, CB2 immunoreactivity increased over neuronal layers as well as in some neuron-free areas being particularly high around Aβ amyloid plaques (Fig 2A, arrow). In neuron-free areas, CB2 signal was low in non-transgenic controls (Fig 2A and 2B).

**Fig 3:**
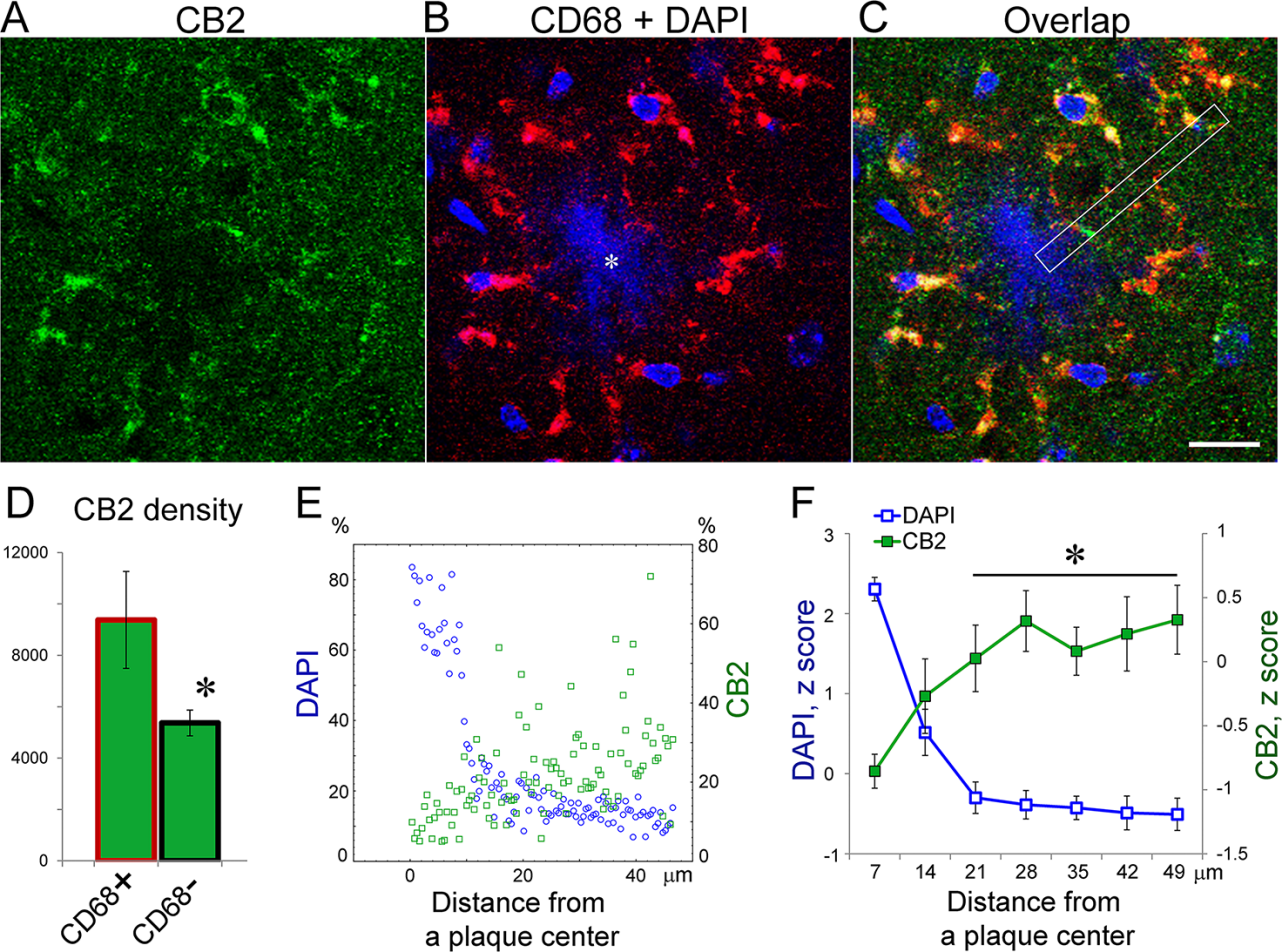
CB2 receptors are expressed in microglial cells and do not accumulate in Aβ plaques of APPswe/PS1ΔE9 mice. A representative confocal image of staining for CB2 receptors (green, H60 antibody) in the cortex of 12 mo-old transgenic mice. (B) An overlap of red (CD68) and blue (DAPI) channels for the image shown in A. Note a characteristic gathering of activated microglia around an amyloid plaque (marked by an asterisk). (C) An overlap of channels shown in A-B. Note that areas with high CB2 intensities overlap with CD68-positive areas. White rectangle shows an example of areas used for quantifications presented in E-F. Scale bar is 15 μm. (D) Quantification of CB2 densities (integrated intensities/area) in CD68-positive and –negative areas. 26 areas like that shown in A-C were used for the quantification (n = 2 transgenic mice). Asterisk indicates a significant difference between CD68+ and CD68- areas (one-way ANOVA, p<0.0001). (E) A scatterplot of CB2 and DAPI intensities as a function of distance from the center of an Aβ plaque with radius ~10 μm. Note low CB2 signal in the core of the plaque. CB2 and DAPI intensities were normalized (%) to a maximum signal on each channel. An example of an area used for calculations is shown by a white rectangle in C. (F) Quantification of CB2 signal at different distances from a plaque center. 4–6 slices of z stacks from five plaques (range of radiuses 7–15 μm) were used in one-way ANOVA. Asterisks indicate a significant increase (p<0.0001, post-hoc test) in CB2 intensities as compared to the core of plaques (radius ≤ 7 μm).

We stained brain slices from 12 mo-old APPswe/PS1ΔE9 transgenic mice with CB2 antibody (H60) and CD68 antibody, a commonly used phenotypic marker for microglia that reflects expression of a lysosomal proteins [59, 60]. Double CB2+CD68 immunostaining revealed prominent CB2 labeling that overlapped with CD68-positive marker (Fig 3A–3C). Double CD68 and CB2-labeled cells showed morphological features of activated microglia, with amoeboid large cell bodies and few thick processes (Fig 3C). Quantitative analysis of CB2 intensities in CD68-positive areas showed a significantly higher signal in microglial cells as compared with CD68-negative areas (Fig 3D).

The core of Aβ amyloid plaques lacked CB2 signal (Fig 3A and 3C). To visualize the core plaques we used co-staining with DAPI instead of typical Thioflavin-S staining, for the reason that latter induced a weak fluorescent signal on more than one channel. 3D reconstruction of DAPI and Thioflavin-S stainings showed that DAPI marks the core of thioflavin-S positive plaques (not shown). Quantification of CB2 signal along the drawing line from the core of DAPI-positive plaques to periphery (Fig 3E) revealed a significant effect of distance from the plaque center (one-way ANOVA, F(1,6) = 42.10, p<0.0001). Plaques used in the analyses had radiuses ranging from ~7 to 15 μm and a minimum of CB2 signal was located in the core common for all plaques (~7 μm radius, Fig 3F).

**Fig 4:**
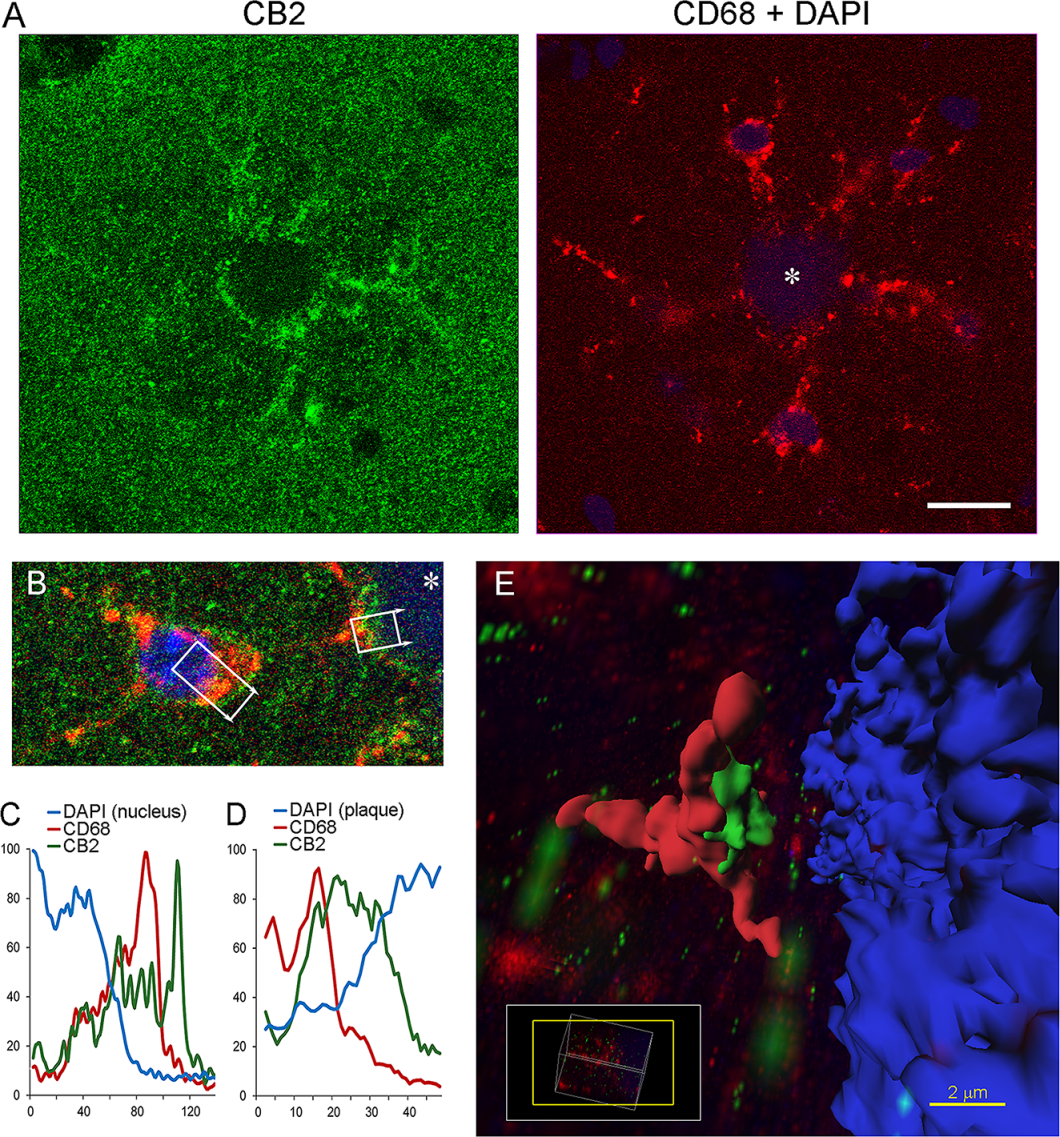
Localization of CB2 receptors in microglial cells and engulfment synapses. (A) A confocal image of CB2 (green) and CD68 staining (red) centered at the core of an Aβ plaque (marked by an asterisk). Note a region of high intensity for CB2 and CD68 staining around the plaque implying that CB2 receptors are localized in microglial processes surrounding the plaque. Scale 15 μm. (B) A magnification of a microglia cell body (red) and its process (red) forming an engulfment synapse on a dense core amyloid plaque (marked by an asterisk, DAPI). Note CB2 receptor staining along the edge of CD68-positive staining and at the engulfment synapse. White boxes indicate areas used for quantifications in C-D. (C-D) Quantification of CB2, CD68, and DAPI signals from the image of a microglia cell body (C) and engulfment synapse (D). The quantification was done using a Plot profile analysis tool (Fiji). Signals were averaged along short axes of boxes shown in B and normalized to a max value (100%) for each channel. Units of X axis are pixels, scale: 11.1 pixels/ μm. (E) 3D reconstruction of the engulfment synapse shown in B, D. A z stack of 0.31 μm slices (n = 29) was processed by using a background subtraction function and normalization for each of the channels. Surfaces for the plaque (DAPI), microglia process (CD68), and CB2 signal were created by arbitrary thresholding at an upper third of intensity distributions. Note high intensities of CB2 signals are located between CD68 and DAPI surfaces. Insert shows orientation of a 3D window as related to a position of the brain slide.

CB2-positive microglial cell bodies and their processes directed toward the core of Aβ plaques formed characteristic star-like structures that were observed throughout different brain structures including the cortex (Fig 3A–3C), hippocampus (Fig 4A), olfactory bulb, and thalamus (not shown). A closer examination of the CB2 staining pattern in microglial cells revealed low signal in the nucleus and higher, easily identifiable, staining in the cytoplasm of the cell body as judged by its overlap with the CD68 lysosomal marker (Fig 4B and 4C). The maximum of CB2 signal in the microglial cell body, however, was located ~1.5 μm to the periphery of the CD68-positive area of the cytoplasm (Fig 4C), implying that some CB2 receptors are likely located on the plasma membrane. The pattern of CB2 staining along microglial processes was more difficult to grasp on 2D plane images. Nevertheless CB2 staining at the end of microglial processes was more robust and detectable in 2D (Fig 4B and 4D). The endings of the microglial processes formed plate–like structures immediately adjacent to the core of Aβ plaques (Fig 4E).

Close proximity of these specialized regions of microglial processes to their intended phagocytic target (plaque) made them analogous to an engulfment synapse observed during an interaction of the phagocyte and apoptotic cell [61]. CB2 receptor immunoreactivity was located inside of such engulfment synapses, mostly at the distal end of CD68-positive microglial processes and in close proximity to the core of a plaque (Fig 4B, 4D and 4E). This pattern of distribution of CB2 receptors created an impression of a CB2-positive halo around Aβ plaques (Fig 4A).

### Comparison of CB2 expression in neurons, microglia, and astrocytes

**Fig 5:**
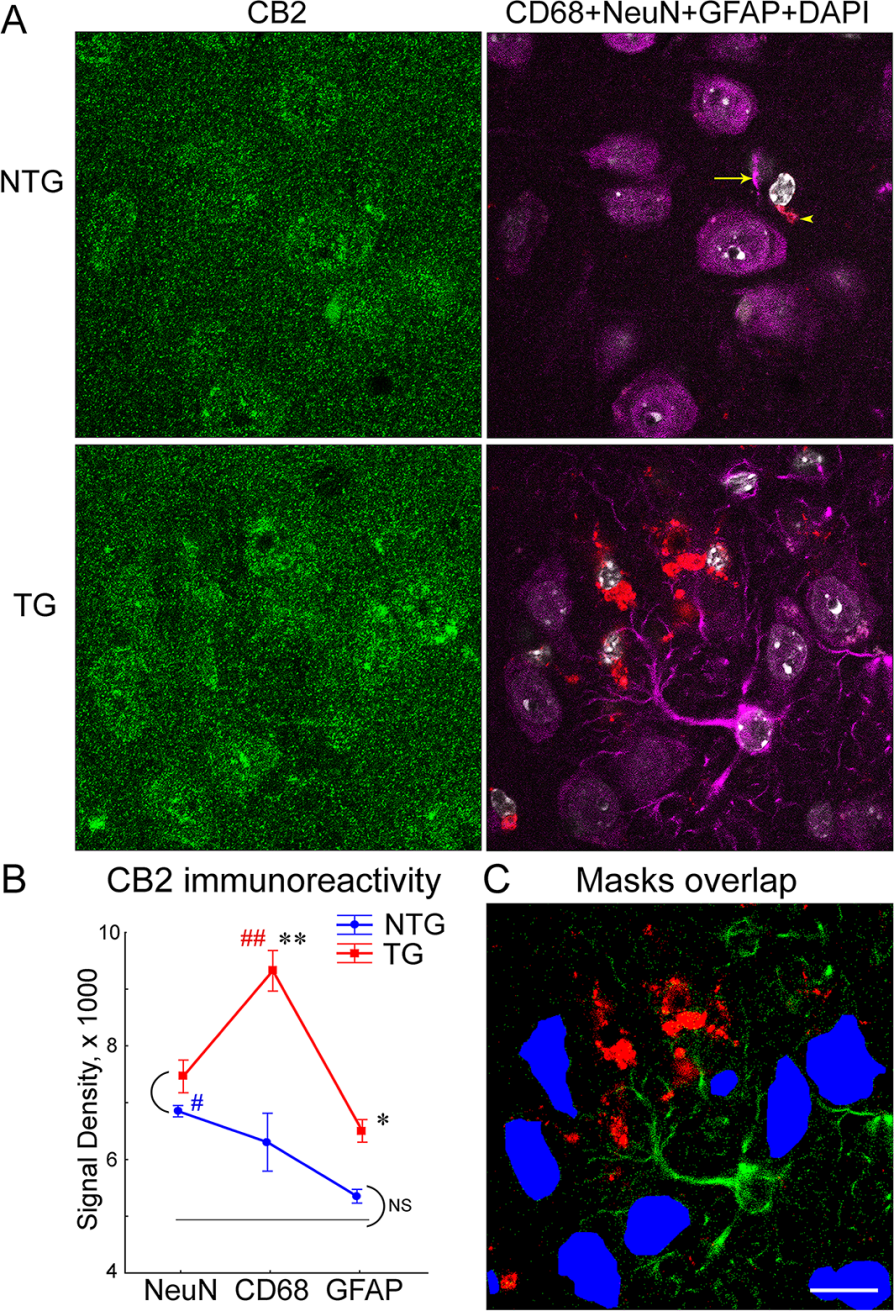
Comparison of CB2 immunoreactivity in neurons, activated microglia and astrocytes. (A) Representative confocal images from the cortex of 12 mo-old non-transgenic (NTG) and APPswe/PS1ΔE9 transgenic (AD) mice stained with a CB2 receptor antibody (H60sc; left columns; green) and markers for neurons (NeuN, far red), activated microglia (CD68, red), and astrocytes (GFAP, far red). Brain slides were counterstained with DAPI shown with a grey pseudo color. Note substantial micro- and astro-gliosis in the cortex of the AD mouse brain. In the NTG mice, CD68+ and/or GFAP+ areas were rare (indicated in the upper right panel by an arrowhead and arrow, respectively). (B) Quantification of densities (+-SEM) for CB2 receptor immunoreactivity (integrated intensities/area) in areas positive for NeuN, CD68, and GFAP markers. Densities were averaged over 22 (AD) and 14 (NTG) images of the cortex as shown in A (n = 2 mice per genotype). Single and double asterisks indicate a significant difference between NTG and AD groups as a result of LSD post-hoc test with p levels <0.01 and 0.0001, respectively. Arcs indicate non-significant (NS) differences. Single and double pound signs (p levels <0.05 and 0.001) indicate markers that correspond to the highest CB2 density in the NTG (blue sign) or AD (red sign) groups (LSD post-hoc test). Solid black line at the level of 4,930 shows average densities for the background. (C) An example of NeuN (blue), CD68 (red), and GFAP (green) masks from the AD image shown in A. NeuN masks were drawn by hand as shown in Fig 2C; masks for CD68 and GFAP were created by a threshold function. Black area represents background. Scale is 15 μm.

The double immunostaining of CB2 receptors with neuronal (NeuN) or microglial (CD68) markers revealed a significantly stronger CB2 signal in neurons and microglia as compared to background (Figs 2, 3 and 4). The additional potential source of CB2 signal could come from astrocytes [11, 62, 63]. To compare CB2 expression in neurons, microglia, and astrocytes we performed co-staining the APPswe/PS1ΔE9 brain slices for CB2 receptor (H60 antibody) with three cellular markers (NeuN, CD68, and GFAP) and DAPI counterstaining for cell nuclei localization. Because only four lasers were available on our confocal microscope, we used the same fluorochrome for NeuN and GFAP labeling. Due to obvious morphological differences between neurons and astrocytes as well as differences in the intensity of GFAP and NeuN signals (Fig 5A) we were able successfully discriminate between these two types of cells (Fig 5C; S3 Fig).

The two-way ANOVA with Genotype as main factor and Cell Marker as repeated measures yielded a significant effect of Genotype (F(1,101) = 47.33, p<0.0001), Cell Marker (F(2,101) = 22.90, p<0.00001) and their interaction (F(2,101) = 9.93, p<0.0005) (Fig 5B). The maximum intensity of the CB2 signal in the NTG mice was originated from neurons (p<0.001 as compared to astroglia, LSD post hoc test). The CB2 signal in the CD68+ ROI was only marginally increased as compared to GFAP+ ROI (p>0.05, LSD post-hoc). Importantly, the CB2 signal derived from astrocytes of the NTG mice was negligible (p>0.23 as compared to the background, one-way ANOVA). These data indicate that the main source of CB2 signal in the cortex of NTG mice derives from neurons, particularly if taking into consideration that CD68 positive areas have been observed in the brains of NTG mice only on very rare occasions (see Fig 5A).

In the APPswe/PS1ΔE9 mice, the maximum intensity of the CB2 signal was observed in microglia (p<0.00001 as compared to astroglia- and neuron-originated signals, LSD post hoc tests). As compared to the NTG littermates, CB2 intensities were significantly increased in microglia as well as astrocytes of the APPswe/PS1ΔE9 mice (p<0.0001 and 0.0002, respectively, one-way ANOVAs). Considering that we didn’t observed increased signal in neurons of the APPswe/PS1ΔE9 mice, these data indicate that glial cells, and CD68-positive microglia in particular, could be the sources of CB2 signals in amyloid-bearing mice.

### Regional distribution and kinetic studies of [^11^C]A836339 in AD and control mice

**Fig 6:**
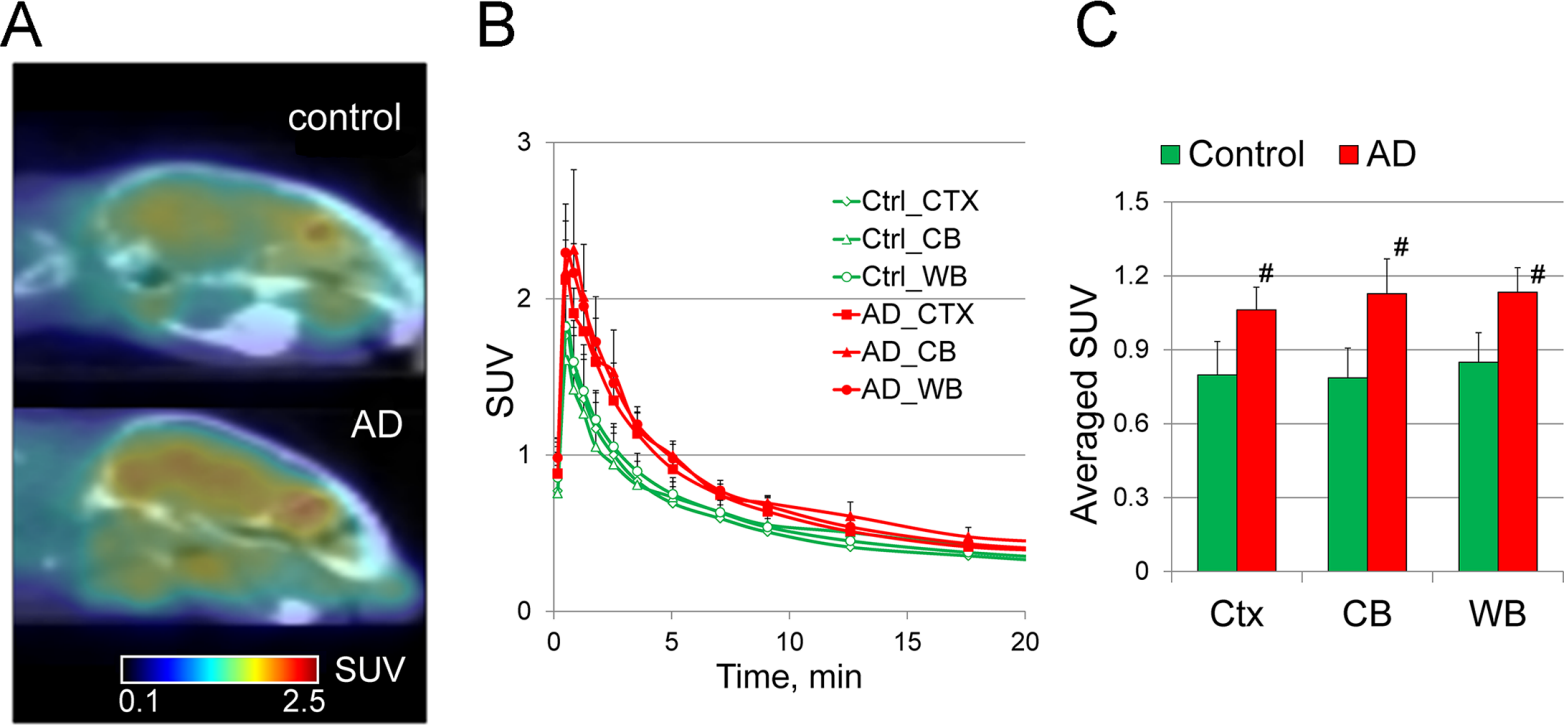
Baseline SUV PET imaging studies of [^11^C]A836339 in AD (APPswe/PS1ΔE9 model) and control non-transgenic mice (mean, n = 3). 12 mo-old male mice were used in this study. (A) Representative sagittal PET images averaged over a period 2–10 min. (B) Brain regional time-uptake curves in AD (red) and control (green) mice. Data: mean SUV ± SD (n = 3 per genotype) (C) Averaged SUV from the same studies. (# *p* ≤ 0.05, ANOVA). Abbreviations: CTX–cortex; CB–cerebellum, WB–whole brain.

Small animal PET sessions were carried out in the APPswe/PS1ΔE9 model. APPswe/PS1ΔE9 transgenic mice and non-transgenic control mice were matched in pairs by sex (males) and age (12 mo of age) and underwent PET scanning simultaneously that allowed to use the same batch of [^11^C]A836339 in both animals. Three pairs of mice were used to test for the effect of group on retention of the tracer (Fig 6). After an i.v. bolus injection, [^11^C]A836339 rapidly entered the mouse brain. The peak accumulation of 2.30 ± 0.20 SUV (mean of 3, ± SD) was reached in the whole brain at 0.5–1 min post-injection, followed by washout to 0.67 ± 0.07 SUV at 10 min and 0.22 ± 0.02 SUV at 20 min (Fig 6C). Time-activity curves in whole brain (WB), cortex (Ctx) and cerebellum (CB) demonstrated comparable kinetics in all three regions.

The [^11^C]A836339 radioactivity accumulation in the control mouse brain was lower than that of APPswe/PS1ΔE9 mice in all three regions studied (WB, Ctx, CB) (Fig 6). These findings are consistent with the pattern of pathology in this AD model in which age-related deposition of Aβ amyloid plaques and concomitant microglial activation is observed throughout the forebrain and cerebellum (S4 Fig). The increase of SUV uptake was significant at 2–10 min post-injection (Fig 6D) and the control-to-AD average SUV difference was greater in the cerebellum (43%) and slightly lower in the cortex and whole brain (33–34%). At the later time points (10–20 min) the difference between the uptake in the cortex of AD and control mice was not significant, mainly due to the spillover effect of radioactivity from non-brain organs in the mouse head. The sagittal baseline PET images of APPswe/PS1ΔE9 and control mouse brain demonstrate the increased uptake in the AD brain and accumulation of radioactivity in the non-brain regions of the mouse head (Fig 6A).

### Blocking with a selective CB2 inverse agonist, AM630

**Fig 7:**
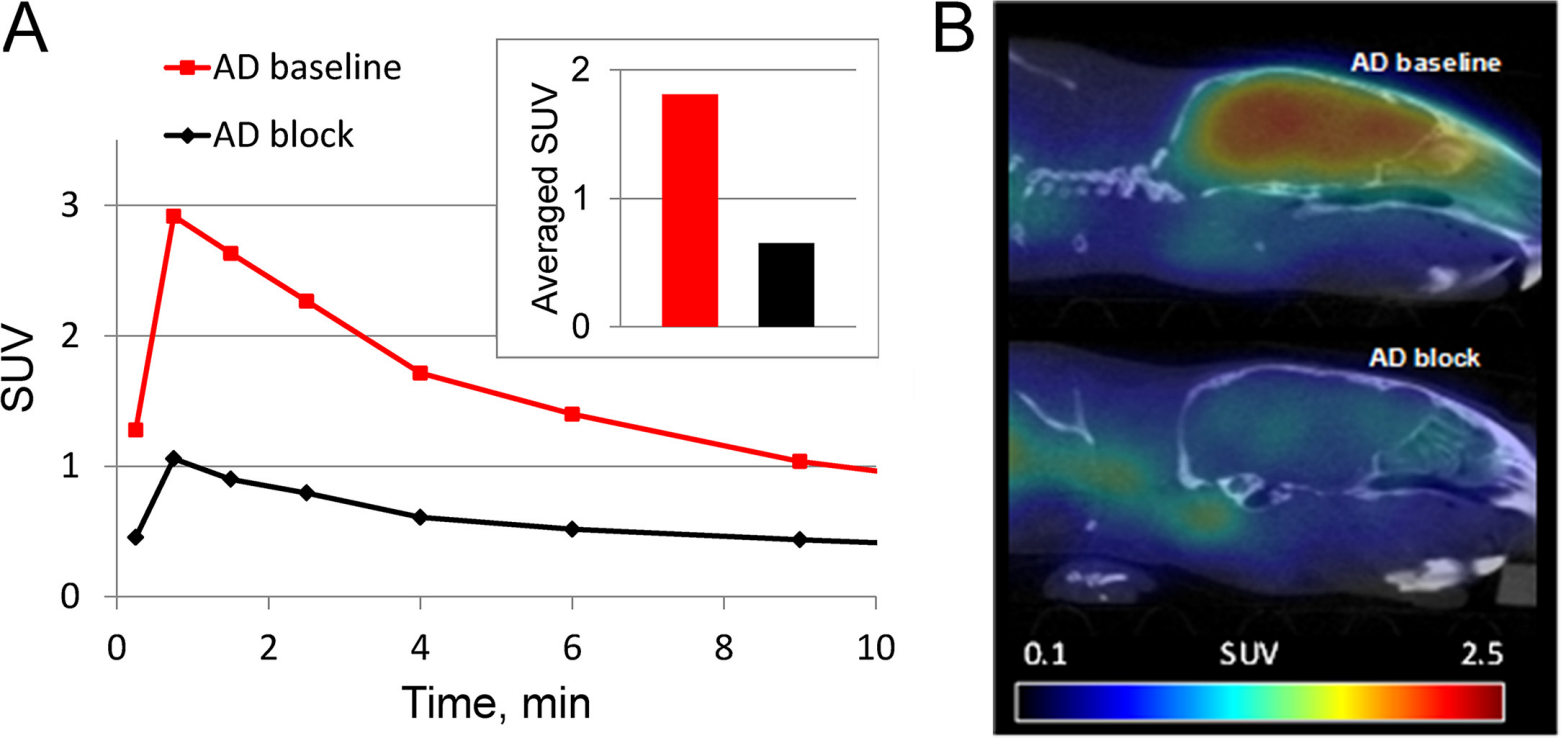
SUV brain PET of [^11^C]A836339 in two AD mice (APPswe/PS1ΔE9) in baseline and blockade experiments with AM-630 (2 mg/kg, s.c.), a selective CB2 inverse agonist. 12 mo-old male mice were used in this study. (A) Whole brain time-uptake curves. Insert: averaged SUV values (2–10 min). Red–baseline; black–blockade. (B) Sagittal baseline (top) and blockade (bottom) images (2–10 min). This study demonstrates that in vivo binding of [^**11**^C]A836339 in AD mice is specifically mediated by CB2 receptors that is consistent with our previous [^**11**^C]A836339 ex vivo studies in AD mice [21].

Previous ex vivo studies demonstrated that [11C]A836339 binding in the AD mouse brain is specific and mediated by CB2 receptors [21]. Here we confirmed those results in vivo by comparison of [11C]A836339 baseline and blockade images in a pair of AD mice (APPswe/PS1ΔE9 model) that were scanned side-by-side. One of the AD mice was scanned after a bolus injection of baseline PET, whereas the other AD mouse was pre-injected with the selective CB2 inverse agonist AM630 [64] before the radiotracer administration. The AD mouse injected with the blocker showed a 2–3 fold reduction of radioactivity uptake in the whole brain (Fig 7) and the averaged SUV value (2–10 min) in the baseline scan was 1.8 versus 0.65 in the blockade scan (Fig 7A).

## Discussion

In the present study we tested whether CB2 receptors were a suitable target for a PET biomarker of neuroinflammation in a mouse model of amyloidosis. In our previous work we used a CB2 radiotracer, [^11^C]A836339, to study regional distribution of CB2 in 18 mo-old APPswe/PS1ΔE9 mice that have Aβ plaque loads comparable to those seen in human cases with advanced AD [21]. That analysis revealed significant specific binding of [^11^C]A836339 in the brain, and particularly, in the cortex [21]. In our present work we demonstrated in PET study that [^11^C]A836339 possessed suitable characteristics, such as CB2-mediated brain uptake and distribution which allow us for discrimination in CB2 retention between 12 mo-old APPswe/PS1ΔE9 mice and their non-transgenic controls.

The analysis of CB2 PET signal from different sub-regions of the brain revealed equally increased uptake of the radiotracer in the cortex and cerebellum. That is an interesting finding, given unequal accumulation of Aβ plaques in these brain regions. Regional dynamics of Aβ accumulation in this mouse model have some similarities to human AD [4], with the hippocampus and cortex being the first structures to show Aβ plaques [30, 65, 66]. The presence of Aβ plaques in the cerebellum of these mice has previously been reported [66, 67] and is consistent with the cerebellar pathology observed in familial AD cases with PS1 mutations [67, 68]. The onset of Aβ accumulation in the cerebellum is 5–6 months later than in the cortex [66, 67]. These characteristics of the model indicate that CB2 PET signal from the cerebellum is induced by much earlier stages of Aβ deposition than in the cortex, supporting the notion that CB2 receptor is suitable as an early marker of Aβ-induced microglial activation.

The detailed immunofluorescence studies of CB2 expression in the APPswe/PS1ΔE9 model elucidate possible sources of the difference in CB2 PET signal in amyloid-bearing vs. control mice. By utilizating double or triple immunostaining with different cellular markers, we were able to observe colocalization of CB2 immunoreactivity in neurons, microglia and astrocytes. The observation of CB2-positive signal in neurons is in agreement with previously reported findings of CB2 expression in a number of brain regions of healthy rodent brain [40, 41, 50, 51]. Importantly, densities of CB2 signal associated with neurons were similar between the APPswe/PS1ΔE9 and control mice. Since this model does not have significant neuronal loss, these data indicate that neuron-derived signals are an unlikely source of the difference seen in CB2 PET. In contrast to the case with neurons, densities of CB2 expression associated with glial, and particularly microglial, markers were significantly higher in the amyloid-bearing mice than in controls.

Presence of CB2 receptors in activated microglia is well documented in AD and numerous models of neuroinflammation [26, 58, 69–71], but existence of CB2 receptors in astrocytes is less acknowledged [11, 62, 63]. In our hands, CB2 signal associated with the astroglial marker was practically negligible in the brains of control mice, while in the APPswe/PS1ΔE9 mice it reached levels significantly higher than background. The amyloidosis-associated increase in the density of CB2 in astroglia was substantially lower than in microglia. Notably, if neuronally-derived CB2 signal had not been excluded from the background, the CB2 signal associated with astroglia would not have been detectable.

A potential source of CB2 signal in amyloid-bearing mice could be CB2 that has passively accumulated in amyloid plaques. It is well known that in addition to Aβ peptides, plaques attract dozens of different proteins such as APP, Apo E, Clusterin, BRI2, LRP etc [72–74]. However, our immunofluorescent studies showed that CB2 receptor immunoreactivity is very low inside Aβ amyloid plaques, making it unlikely that there is any significant accumulation of inert CB2 within plaques.

These data allow for the conclusion that the main source of CB2 PET signals in APPswe/PS1ΔE9 mice as compared to controls is an increase in CB2 density associated with activated microglia.

How our preclinical studies with the CB2 radiotracer relate to its potential role as a marker of neuroinflammation in AD is a matter of future clinical trials. The presence of CB2 immunostaining in both neurons and glia suggests that increased CB2 PET signal retention in APPswe/PS1ΔE9 mice could reflect total signal coming from both types of cells. In a situation where there is no neuronal loss (as in the APPSwe/PS1ΔE9 mice), the difference in [^11^C]A836339 retention between control and amyloid-bearing mice would likely reflect CB2 receptor activation on microglia. It is possible to speculate that significant neuronal loss could decrease CB2 PET signal due to loss of neuronally-derived CB2 receptor. Recently, a group from Leuven (Belgium) conducted a small clinical trial with a CB2 tracer, [^11^C]NE40 [18] and reported no differences in the [^11^C]NE40 binding potential between AD patients and age-matched controls. One possible explanation for those findings could be the advanced stage of the disease progression in AD patients involved in the study, as they characterized by significant brain atrophy, associated with a decreased number of neurons. It is possible that utilization of a CB2 tracer with higher binding affinity might increase the likelihood for detection of differences between AD patients and controls. For example, in a CB2 *in vitro* binding assay under the same conditions, our [^11^C]A836339 demonstrated higher binding affinity (*K*_i_ = 0.75; 1.6 nM) than that of [^11^C]NE40 (*K*_i_ = 5.7; 12 nM), suggesting that [^11^C]A836339 may exhibit higher specific binding *in vivo*. However, if CB2 PET signal reflects both microglial and neuronal CB2 receptors, an improvement in radiotracer binding affinity will not necessarily translate to a more accurate measure of microglial activation if used in a setting of significant neuronal loss. Indeed, the Leuven investigators explained their failure to observe a difference between AD patients and controls by possible binding of CB2 tracer to neuronally-derived CB2 receptor. That suggestion is in agreement with our preclinical findings and implies that a CB2 radiotracer should be used in patients at the early stages of AD when no significant neuronal loss has being developed yet.

Another interesting finding was the presence of CB2 receptors in engulfment synapses. At smaller resolution, a halo of increased CB2 density was evident around the plaques due to CB2 presence in the microglial processes contacting the plaque. Such distribution is in agreement with observations in human AD demonstrating that CB2 is abundantly expressed in microglia [26] and is associated with Aβ deposition [58]. In our study, 3D reconstruction of the microglial processes in close vicinity to Aβ plaques demonstrated plate- or hand-like structures analogous to an engulfment synapse, a structure where specialized areas of microglial processes come into contact with an intended phagocytic target [61, 75, 76]. Maximal densities of CB2 receptors were observed between picks of CD68 and DAPI markers, implying localization of CB2 receptors at the plasma membrane of microglia forming the engulfment synapse with fibrillar Aβ. The functional significance of CB2 receptors at the engulfment synapse requires further study. These findings suggest that CB2 receptors are abundant in such synapses, consistent with the role of these receptors in phagocytosis. *In vitro* data documented that pharmacological activation of CB2 receptors on human macrophages resulted in removal of Aβ plaques from human AD brain sections [71]. The stimulation of CB2 has also been shown to increase capacity of microglial cells to phagocytize Aβ1–42 peptide [77]. Recent *in vivo* studies in animal models of AD demonstrated an ameliorating effect of CB2 receptor agonists on cognitive deficits in these mice that was associated with decreased microglial reactivity, reduced expression of pro-inflammatory cytokines [78, 79] and some decreases in Aβ accumulation after longer periods of treatments [79]. Genetic ablation of CB2 receptors in amyloid-producing mice resulted in opposing but significant effects on Aβ plaque load in different models [80, 81] indicating that effects of CB2 receptors is likely to depend of the stage of amyloidosis and other factors.

### Conclusion

Our study demonstrated that Aβ amyloidosis without concomitant tau pathology is sufficient to activate CB2 receptors that are suitable as an imaging biomarker of neuroinflammation in this condition. In particular, the CB2 PET radiotracer [^11^C]A836339 can be used as an imaging biomarker of neuroinflammation in a model of AD. Immunofluorescent analysis indicated that the main source of the difference in CB2 PET signal between amyloid-bearing and control mice was due to CB2 expression in glial cells, and activated microglia in particular. Amyloid-bearing mice had neuronal CB2 densities similar to controls. These data in addition to a lack of significant neuronal loss in this model imply that neuronal CB2 is not likely to contribute to the enhanced CB2 PET signal. However, it is conceivable that significant loss of neurons as at later stages of AD might decrease neuron-derived CB2 signal and blur the differences between controls and AD subjects. This scenario has to be tested in future studies to define stages of disease progression in which sensitivity of CB2 as a biomarker of neuroinflammation is not affected by significant loss of neurons.

## Supporting Information

S1 Fig CB2 immunoreactivity in neurons of CA3 area of the hippocampus of 12 mo-old APPswe/PS1ΔE9 transgenic mouse. Representative confocal images of double immunostaining using NeuN (green; **A**) and CB2 R (red, H60 Santa Crus antibody; **B**) primary antibodies. The section was also counterstained with DAPI (not shown). Panel **C** shows an example of hippocampal neuron (inserts in A-B). C1 shows CB2 immunostaining (red) with neuronal border. Note autofluorescent cytoplasmic granules (pseudo-yellow in c1-c2) derived from overlap of NeuN and DAPI channels (c2). Scale in A-B is 15 μm.
(TIF)

S2 Fig CB2 immunoreactivity in neurons of the cortex of 12 mo-old APPswe/PS1ΔE9 transgenic mice as revealed by N-terminal CB2 antibody (Cayman # 101550). Representative confocal images of double immunostaining using NeuN (green) and CB2 R (red) primary antibodies. The section was also counterstained with DAPI (not shown). Cayman CB2 antibody revealed enhanced staining in the cytoplasm of neurons. Also note good tracing of neuronal processes with this CB2 antibody (arrows). Scale is 15 μm.
(TIF)

S3 Fig Discrimination of neurons and astroglia by using the same fluorochrome. To compare CB2 expression in neurons, microglia, and astrocytes we performed co-staining of the APPswe/PS1ΔE9 brain slices for CB2 receptor (H60 antibody) with three cellular markers (NeuN, CD68, and GFAP) and DAPI counterstaining for cell nuclei localization (Fig 5). Because only four lasers were available on our confocal microscope, we used the same fluorochrome for NeuN and GFAP labeling. Discrimination between neuronal and astroglial CB2 signal was based on morphological differences between neurons and astrocytes as well as differences in the intensity of GFAP and NeuN signals (Fig 5A). To analyze the rate of errors introduced by using the same fluorochrome for labeling neurons and astroglia, we performed additional staining of brain slices from 12 mo old APPswe/PS1ΔE9 mice. mouseNeuN and mouseGFAP primary antibodies (the same as in Fig 5) were visualized by a single fluorochrome, and signal was digitized through a red channel. Another GFAP primary antibody (rabbitGFAP) was visualized by a different fluorochrome, and signal was processed through a green channel. DAPI was used for labeling nuclei (blue channel). **A**. Representative confocal image with neuronal marker (NeuN, red fluorochrome), astroglial marker (Sigma mGFAP; red fluorochrome) is shown as a composite with DAPI (blue). Outlines of neurons are shown by yellow lines. **B**. The same area as in A with another astroglial marker (Dako rbGFAP; green fluorochrome). The outlines of neurons are transferred from A. Note that cases of overlap between neuronal outlines and the astroglial marker with separate fluorochrome are very rare. **C**. Pie chart of the rate of errors discriminating neurons and astroglia. rbGFAP signal (green fluorochrome, as in B) was filtered (Gaussian Blue) and binarized (IsoData threshold). An overlap between rbGFAP-positive area and neuronal outlines (as in A) was calculated for total of 21 images from 2 mice. The rate of error (0.9±0.5%) is expressed relative to total neuronal area. **D-E**. Examples of binarized (IsoData threshold) signals from mGFAP (red fluorochrome) and rbGFAP (green fluorochrome). D also show neuronal outlines (white lines) based on NeuN staining using the same fluorochrome as for mGFAP. Green indicates areas with rbGFAP signal and yellow shows areas of overlap between rbGFAP and mGFAP signals. Red indicates areas of mGFAP signal that were not confirmed by rbGFAP antibody (see one of the examples pointed out by an arrow in D). **F**. Pie chart showing percent of mGFAP signal on the channel with dual astroglial and neuronal staining that was not confirmed by rbGFAP signal on a separate channel. The total of 31 images from 2 mice was processed as described in C. The rate of error (4.9±3.0%) is expressed relative to total mGFAP area. Scale in A-B, D-E is 10 μm.
(TIF)

S4 Fig Presence of Aβ amyloid plaques in the cortex, hippocampus and cerebellum of APPswe/PS1ΔE9 transgenic (Tg) mice. Representative examples of Thioflavin-S staining are shown for 11 and 18 mo-old male transgenic mice. Scale is 250 μm.
(TIF)
