# Supplementary figures and images for: Cannabinoid CB2 Receptors in a Mouse Model of Aβ Amyloidosis: Immunohistochemical Analysis and Suitability as a PET Biomarker of Neuroinflammation
Source: PLoS One. 2015 Jun 18;10(6):e0129618. doi: 10.1371/journal.pone.0129618 (PMC4472959; doi:10.1371/journal.pone.0129618)

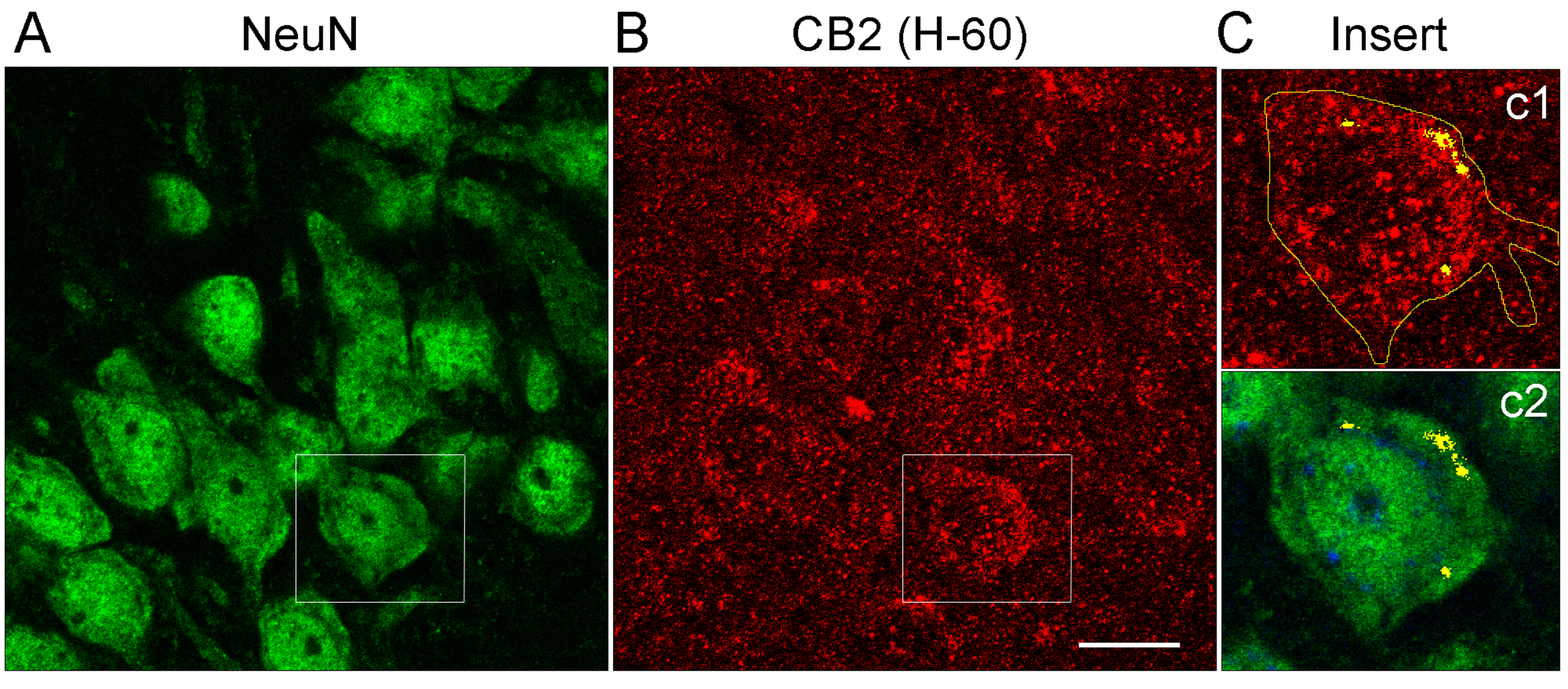

Supplement: S1 Fig — Representative confocal images of double immunostaining using NeuN (green; A) and CB2 R (red, H60 Santa Crus antibody; B) primary antibodies. The section was also counterstained with DAPI (not shown). Panel C shows an example of hippocampal neuron (inserts in A-B). C1 shows CB2 immunostaining (red) with neuronal border. Note autofluorescent cytoplasmic granules (pseudo-yellow in c1-c2) derived from overlap of NeuN and DAPI channels (c2). Scale in A-B is 15 μm. (TIF) [file pone.0129618.s001.tif]

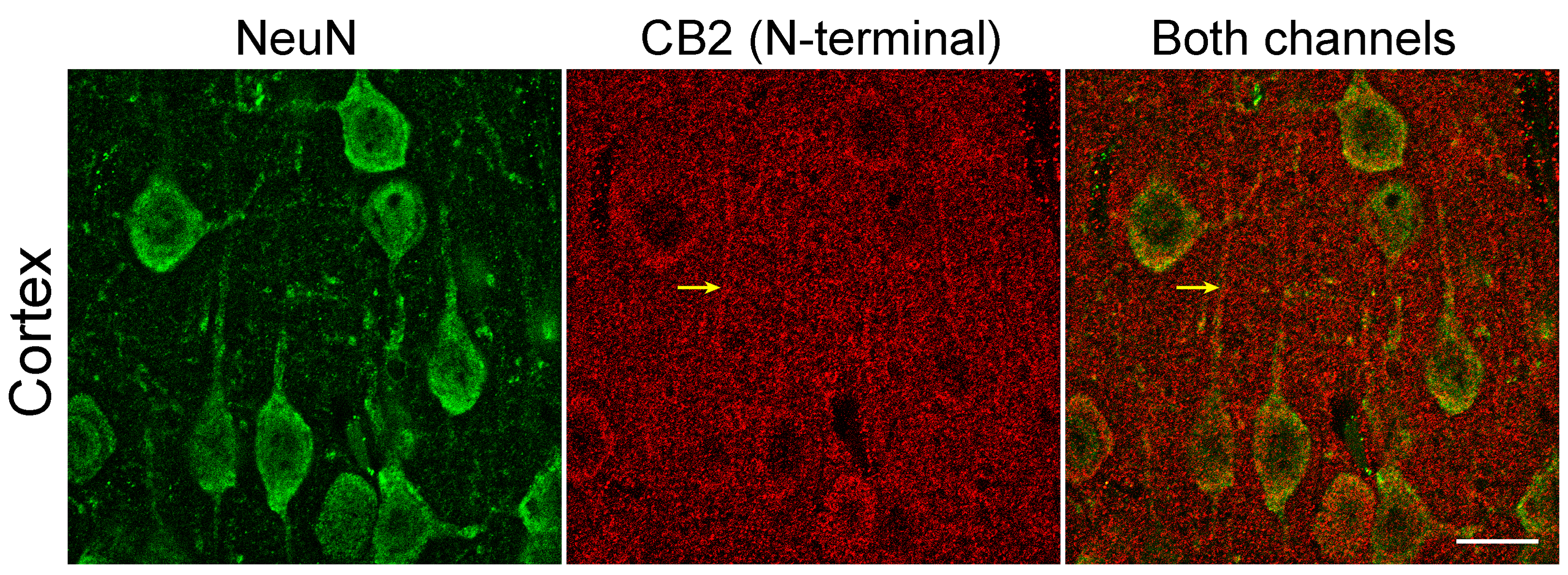

Supplement: S2 Fig — Representative confocal images of double immunostaining using NeuN (green) and CB2 R (red) primary antibodies. The section was also counterstained with DAPI (not shown). Cayman CB2 antibody revealed enhanced staining in the cytoplasm of neurons. Also note good tracing of neuronal processes with this CB2 antibody (arrows). Scale is 15 μm. (TIF) [file pone.0129618.s002.tif]

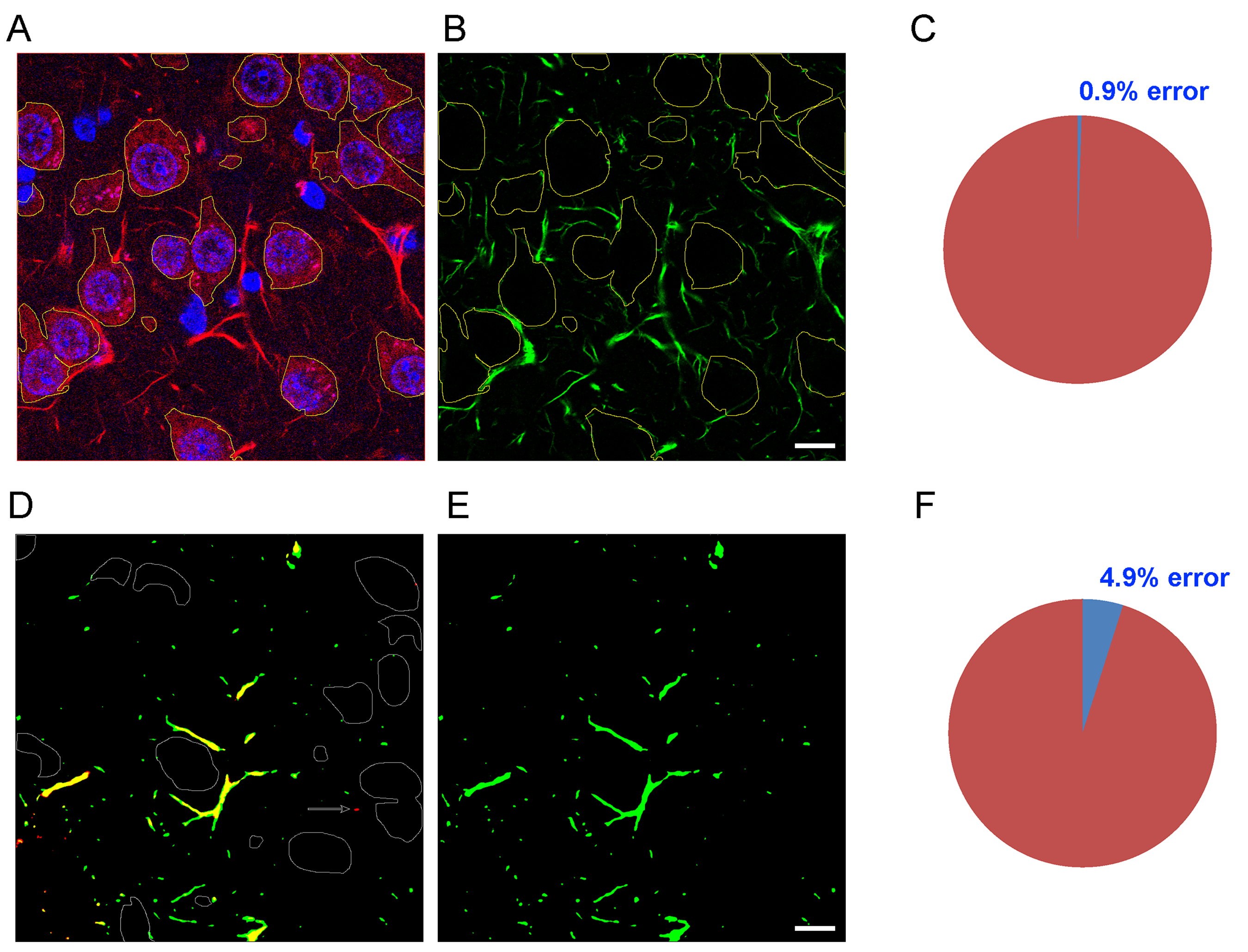

Supplement: S3 Fig — To compare CB2 expression in neurons, microglia, and astrocytes we performed co-staining of the APPswe/PS1ΔE9 brain slices for CB2 receptor (H60 antibody) with three cellular markers (NeuN, CD68, and GFAP) and DAPI counterstaining for cell nuclei localization (Fig 5). Because only four lasers were available on our confocal microscope, we used the same fluorochrome for NeuN and GFAP labeling. Discrimination between neuronal and astroglial CB2 signal was based on morphological differences between neurons and astrocytes as well as differences in the intensity of GFAP and NeuN signals (Fig 5A). To analyze the rate of errors introduced by using the same fluorochrome for labeling neurons and astroglia, we performed additional staining of brain slices from 12 mo old APPswe/PS1ΔE9 mice. mouseNeuN and mouseGFAP primary antibodies (the same as in Fig 5) were visualized by a single fluorochrome, and signal was digitized through a red channel. Another GFAP primary antibody (rabbitGFAP) was visualized by a different fluorochrome, and signal was processed through a green channel. DAPI was used for labeling nuclei (blue channel). A. Representative confocal image with neuronal marker (NeuN, red fluorochrome), astroglial marker (Sigma mGFAP; red fluorochrome) is shown as a composite with DAPI (blue). Outlines of neurons are shown by yellow lines. B. The same area as in A with another astroglial marker (Dako rbGFAP; green fluorochrome). The outlines of neurons are transferred from A. Note that cases of overlap between neuronal outlines and the astroglial marker with separate fluorochrome are very rare. C. Pie chart of the rate of errors discriminating neurons and astroglia. rbGFAP signal (green fluorochrome, as in B) was filtered (Gaussian Blue) and binarized (IsoData threshold). An overlap between rbGFAP-positive area and neuronal outlines (as in A) was calculated for total of 21 images from 2 mice. The rate of error (0.9±0.5%) is expressed relative to total neuronal area. D-E. Exam [file pone.0129618.s003.tif]

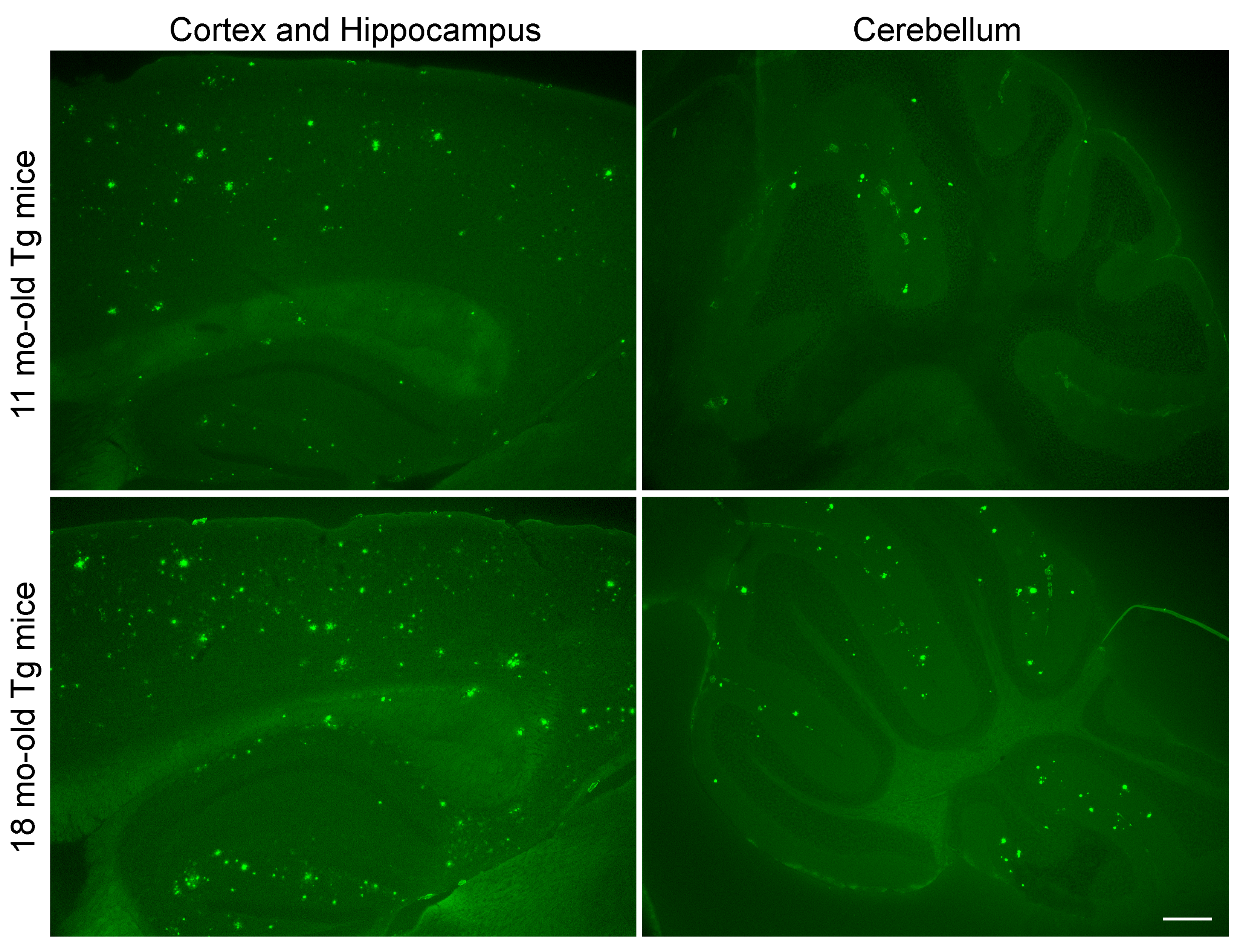

Supplement: S4 Fig — Representative examples of Thioflavin-S staining are shown for 11 and 18 mo-old male transgenic mice. Scale is 250 μm. (TIF) [file pone.0129618.s004.tif]
